# Boosted acceleration of protons by tailored ultra-thin foil targets

**DOI:** 10.1038/s41598-019-55011-2

**Published:** 2019-12-10

**Authors:** Vural Kaymak, Esin Aktan, Mirela Cerchez, Bentsian Elkin, Marc Papenheim, Rajendra Prasad, Alexander Pukhov, Hella-C. Scheer, Anna-Marie Schroer, Oswald Willi, Bastian Aurand

**Affiliations:** 1 Institut für Theoretische Physik I, Heinrich-Heine-Universität, 40225 Düsseldorf, Germany; 2 Institut für Laser- und Plasmaphysik, Heinrich-Heine-Universität, 40225 Düsseldorf, Germany; 3 Fraunhofer Institute for Interfacial Engineering and Biotechnology IGB, 70569 Stuttgart, Germany; 4 Fakultät für Elektrotechnik, Informationstechnik, Medientechnik, Bergische Universität Wuppertal, 42119 Wuppertal, Germany

**Keywords:** Laser-produced plasmas, Plasma-based accelerators

## Abstract

We report on a detailed experimental and numerical study on the boosted acceleration of protons from ultra-thin hemispherical targets utilizing multi-Joule short-pulse laser-systems. For a laser intensity of 1 × 10^20^ W/cm^2^ and an on-target energy of only 1.3 J with this setup a proton cut-off energy of 8.5 MeV was achieved, which is a factor of 1.8 higher compared to a flat foil target of the same thickness. While a boost of the acceleration process by additionally injected electrons was observed for sophisticated targets at high-energy laser-systems before, our studies reveal that the process can be utilized over at least two orders of magnitude in intensity and is therefore suitable for a large number of nowadays existing laser-systems. We retrieved a cut-off energy of about 6.5 MeV of proton energy per Joule of incident laser energy, which is a noticeable enhancement with respect to previous results employing this mechanism. The approach presented here has the advantage of using structure-wise simple targets and being sustainable for numerous applications and high repetition rate demands at the same time.

## Introduction

The acceleration of protons and ions by the interaction of high-intensity laser pulses with matter became a well established field since the first experiments in the 1980s^1^ and early 2000s^2,3^. This all-optical approach capable of reaching tens of megaelectronvolt (MeV) of kinetic energy over a sub-mm length with low-emittance, high-laminarity^4^ and ultra-short duration^5^ holds promise for numerous applications within the field of physics but as well for applications. A detailed review on laser-based ion acceleration and its applications can be found e.g. in the reviews by *Macchi et al*.^6,7^. The most studied process, the so-called target normal sheath acceleration (TNSA)^8,9^ creates a broad energy spectrum particle beam, following a thermal distribution up to a certain cut-off energy (*E*_cutoff_) which scales with ${E}_{{\rm{cutoff}};{\rm{TNSA}}}\propto {I}_{0}^{0.5}$^10^, where *I*_0_ is the laser intensity. Based on this scaling, higher laser intensities lead to higher particle energies, with the drawback that every Joule of additional laser energy comes with a nonlinear increase of investment cost. This can only be realized in a very few international projects, like the upcoming European-Light-Infrastructure (ELI) facilities^11^. Therefore, it is of vital importance, especially for the numerously used class of terawatt laser-systems operated in many laboratories around the world, to have alternative routes to access higher ion energies. Consequently, investigations have addressed, among others, the influence of the target thickness^12^, laser pulse duration^13^ and plasma gradients^14^. Another extensively studied subject is the modification of the target using different methods, in order to obtain an increase of the hot electron sheath population responsible for the accelerating TNSA field.

A different route to reach higher particle energies is the use of acceleration schemes like radiation pressure acceleration (RPA)^15,16^. This attracted attention due to the promising properties such as a proton cut-off energy scaling of ${E}_{{\rm{cutoff}};{\rm{RPA}}}\propto {I}_{0}$^10^ and its expected high energy conversion efficiency at ultra-relativistic intensities^17^. While the fundamental RPA process benefits from the use of circular polarized light^18^, it was demonstrated that the use of a linear polarized light at high intensities leads to a hybrid acceleration regime, in which TNSA and RPA coexist^19–21^. It has been numerically shown to surpass the RPA^22^ despite its expectedly lower energy scaling somewhere in between TNSA and RPA. The highest ion energy achieved so far in an experiment by a laser-driven source of about 94 MeV^23^ has been produced in this hybrid regime.

Here we report for the first time in a detailed experimental and numerical study using a combination of an ultra-thin and tailored target, irradiated by a linearly polarized femtosecond laser pulse at an intensity of 1 × 10^20^ W/cm^2^. We observe an enhanced proton acceleration compared to the classical TNSA scheme and the transition into the hybrid regime of TNSA and RPA towards higher intensities. Using PIC simulations we identified a mechanism in which additional electrons extracted from the target front side are supplied to the hot electron population at the rear side, which was called direct laser light pressure acceleration (DLLPA) observed for μm-thick targets before^24^. We investigate for the first time in detail the amplification of the longitudinal accelerating electric field and the influence of the laser polarization. The DLLPA scheme in combination with ultra-thin tailored targets shows to reliably enhance ion energies over a wide range of intensities and an energy scaling of ${E}_{{\rm{cutoff}}}\propto {I}_{0}^{0.73}$ is evaluated, which represents an improvement with regard to the classical TNSA and lies in the expected range for the hybrid regime. About 70 MeV of proton energy is expected by using only 18 J of laser energy, which suggests that smaller laser-systems can benefit from this method as it indicates an enabled access to ion energies as high as those produced by larger laser facilities while reducing the necessary laser input energies.

## Setup

**Figure 1:**
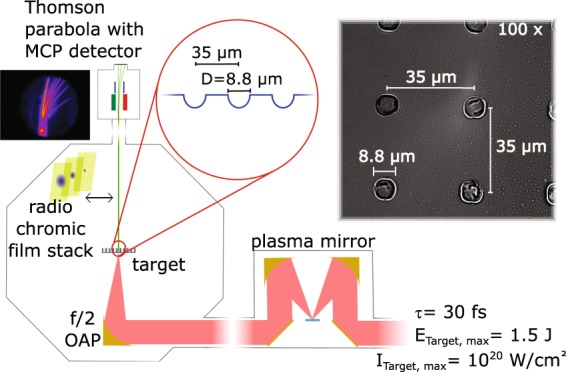
The experimental setup is shown. The contrast of the laser pulse is improved by a plasma mirror before it impinges onto the target under normal incidence. As diagnostic a Thomson parabola spectrometer equipped with an MCP readout system for energy measurements, or alternatively a stack of radiochromic films for reconstruction of the spatial beam profile, is used. (Inset picture) Microscope image of the 110 nm-thick free-standing target used for the experiment. The homogeneous pattern of the hemispheres is visible.

The experiment was carried out at the ARCTURUS 200-TW laser-system of the University of Düsseldorf, which is based on a double CPA Ti:sapphire architecture with a pulse duration of 30 fs and a pulse energy of up to 7 J before compression^25^. The initial laser contrast, which is better than 1:10^−8^ at 80 ps before the main pulse, is further enhanced by another three orders of magnitude using a single plasma mirror based on the design of ref. ^26^. The experimental arrangement is shown in Fig. 1. The 100 mm diameter beam was focussed by an *f*/2 off-axis parabolic mirror (OAP) under normal incidence onto the target. A free-standing parylene-foil (C_8_H_8_) with a thickness of *d* = 110 nm imprinted with a pattern of spherical hollow half shells (hemispheres) of the same thickness (Fig. 1 inset) was used. Those shells were measured to have a diameter of *D* = (8.8 ± 1.2) μm, separated by a centre-to-centre distance of 35 μm from each other. For all studies the laser was incident onto the convex side of the hemispheres.

For the laser intensities and target thicknesses we used, the influence by the formation of a pre-plasma needs to be considered. On this topic several studies have been done^27,28^. Comparing especially with a model from ref. ^28^ with contrast measurements of the laser-system used, reveals that the influence of pre-plasma can be neglected. In addition, we verified, using thinner diamond-like-carbon (DLC) targets, that the resulting proton cut-off energy is stable which is an experimental indicator for a sufficient laser contrast.

Additional information on the target production, characteristics as well as the experimental diagnostics can be found in the methods section.

## Experimental Results

The laser beam was focussed to a diameter of 7 μm (FWHM), corresponding to a focal spot size of 40 μm^2^. The on-target energy of 1.3 J gives an intensity of 1 × 10^20^ W/cm^2^, equivalent to a normalized vector potential of ${a}_{0}=6.8$. The target was positioned and verified to be in focus before each shot. Note that the pointing stability of the laser-system was measured to be (2.1 ± 3.1) μm over 30 minutes. Aligning always onto the center of the hemisphere and taking the focus diameter as well as the pointing-stability into account it can be assumed that the structure was fully or at least partially irradiated on every shot.

**Figure 2:**
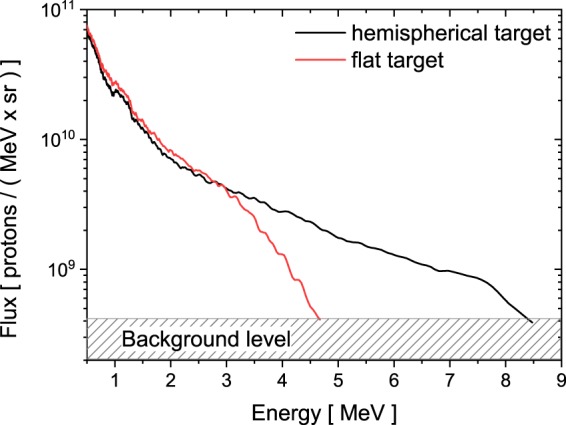
Proton energy spectra recorded by a Thomson parabola in laser forward direction for ${a}_{0}=6.8$.

The energy spectra were measured by a Thomson parabola spectrometer in forward direction (Fig. 2), for which a cross-calibration with CR-39 nuclear track detector was done to provide the absolute proton flux^29^. A typical energy resolution of about 10% is retrieved taking the pinhole size into account.

For the flat foil a proton cut-off energy of 4.6 MeV was achieved, while with the same laser parameters incident on the hemispherical structures an energy of 8.5 MeV was measured. This corresponds to an energy gain by a factor of 1.8 by the hemispherical target.

**Figure 3:**
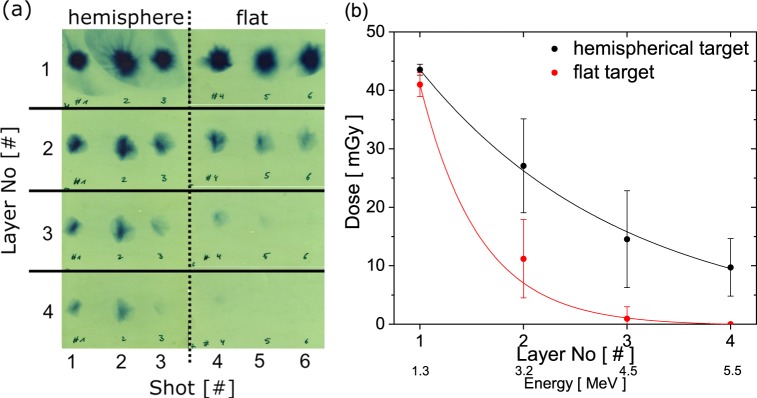
(**a**) Raw image of a four-layer RCF stack for three shots each recorded on hemispherical- and flat targets. (**b**) Evaluated dose, depending on the layer number/energy for these two cases.

Figure 3(a) shows the raw image of three consecutive laser shots on the hemispherical structures and three consecutive shots on the flat target positions in between, recorded by radiochromic film (RCF). The evaluation of the absolute dose is given in Fig. 3(b), using a cross-calibration of the particular RCF batch done at a cyclotron. Here the error bars denote the standard deviation based on the three individual shots for both cases.

The evaluation of the dose revealed significantly more high energetic protons from the hemispherical target compared to the flat target. This finding is in agreement with the energy spectra obtained by the Thomson parabola in Fig. 2 for both targets.

## Numerical Simulation and Discussion

**Figure 4:**
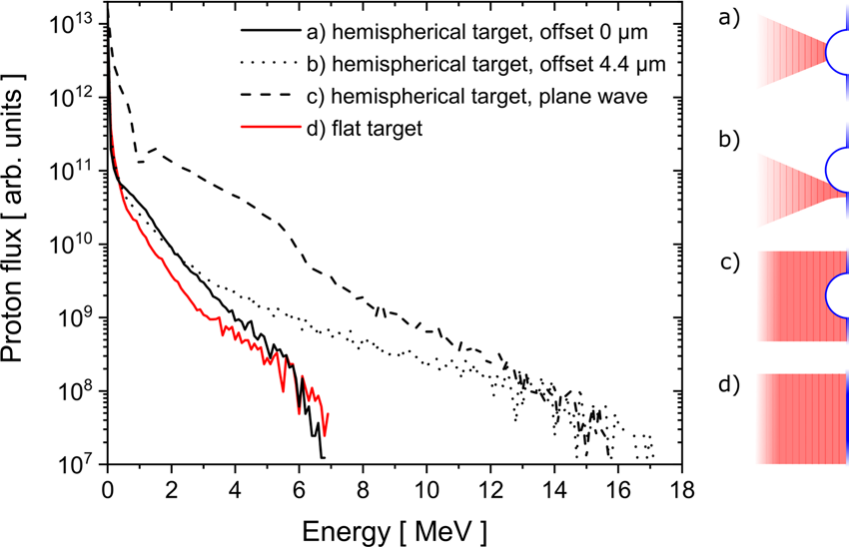
Integrated proton spectra obtained from the simulations for a flat (red line) and a hemispherical target (black line) at ${a}_{0}=6.8$, for different irradiation positions relative to the centre of the hemispherical structure, as illustrated on the right hand side. All spectra show a TNSA like particle distribution. The cut-off energy is enhanced by the structured target up to a factor of up to 2.1 compared to a flat foil.

**Figure 5:**
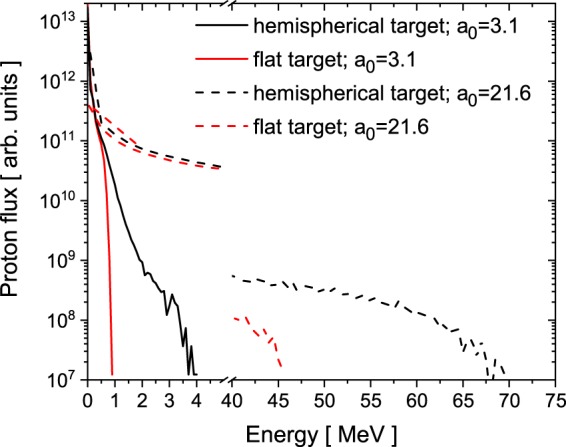
Computed proton spectra for intensities 2 × 10^19^ W/cm^2^ (${a}_{0}=3.1$) and 1 × 10^21^ W/cm^2^ (${a}_{0}=21.6$) as an extrapolation from the experimental parameters.

To investigate the cause of the energy gain by the tailored targets, numerical simulations using the PIC code VLPL^30,31^ were carried out. To retrieve the numerical time integrated energy spectra of the protons (Figs. 4 and 5), all particles that move away from the back of the foil were added up, whereby information on particles leaving the simulation box were stored and also taken into account. Modelling the experimental conditions at an intensity of 1 × 10^20^ W/cm^2^ (${a}_{0}=6.8$) the achieved cut-off energy with the flat target is about 7.0 MeV (Fig. 4).

For the hemispherical target three simulations were conducted. The first, with the focussed pulse hitting the centre of the hemisphere, another one for which the focus is shifted by the hemisphere radius of 4.4 μm in polarization direction *y*, hitting the edge of the hemisphere, and a third one with a plane wave (Fig. 4). It turns out that the irradiation position plays an important role as the focal spot diameter (7 μm) is comparable to the hemisphere diameter (8.8 μm).

While the on-axis pulse does not yield higher energies compared to the flat target, one finds proton energies of 15–17 MeV for the case of a shifted pulse and a plane wave, which denotes an energy enhancement factor larger than 2.1 for the hemispherical target compared to the flat foil. Note, that the plane wave gives rise to a higher number of particles along the lower energy range, owed to the TNSA taking place across the whole target, in contrast to the irradiation of a finite area as in the case of a focussed pulse. From this special cases, it can be deduced that cut-off energies between 7 MeV and 15 MeV are expected in the experiment.

**Figure 6:**
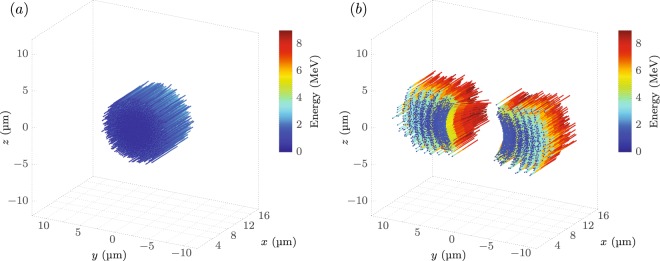
(**a**) Trajectories of the protons that reach energies of at least 2.0 MeV, using a focussed laser pulse with an intensity of ${a}_{0}=6.8$ and a focal spot of 7 μm at FWHM irradiating a flat C_8_H_8_ foil of $d=110\,{\rm{nm}}$ thickness. (**b**) Trajectories of protons reaching at least 7.5 MeV, using a plane wave with ${a}_{0}=6.8$ irradiating a C_8_H_8_ foil of $d=110\,{\rm{nm}}$ thickness with a hemispherical hollow shell of 8.8 μm diameter. The laser pulse in (**a**,**b**) has a wavelength of ${\lambda }_{0}=800\,{\rm{nm}}$, pulse duration of *t*_L_ = 35 fs and enters the simulation box at $x=0$.

As experiment and numerical simulations point out, there is an increase in the higher energetic particles, visible as an elongation of the tail above the cut-off energy of the flat target. This is an indication that a part of the protons gain more energy in the case of a hemispherical target. It remains to be clarified what the cause is and where those protons originate from. For a further investigation the trajectories and energies of protons with >7.5 MeV (hemispherical target) and >2 MeV (flat target) were recorded during the full simulation in order to identify where exactly those high energy particles emerge from. In all following illustrations regarding the hemispherical target a plane wave simulation was chosen for the sake of symmetry, as physics-wise the results do not differ from the focussed pulse case. Figure 6 shows a projection of those trails, while the colour encodes the particle energy.

For the flat target [Fig. 6(a)], the particles move normal to the surface and parallel to each other, with a homogeneous energy gain distributed over the laser focal spot. In the case of the hemispherical target [Fig. 6(b)] the particles that have a higher energy than in the flat target case (compare in Fig. 4) are depicted. They mainly stem from an about 5 μm wide flat foil area at the sides of the hemisphere along the laser polarization direction and move normal to the target. The reason behind this observation implies two modifications to the classical TNSA acceleration scheme and the evolution of the electron sheath field.

**Figure 7:**
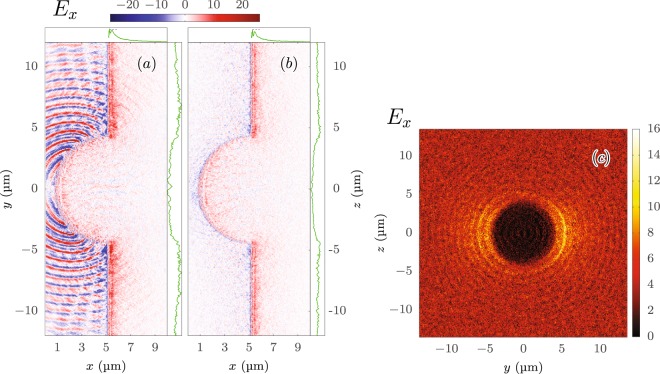
Cross sections of the electric field component *E*_*x*_ [TV/m] normal to the surface in (**a**) the plane of polarization (*y*–*x*), (**b**) perpendicular to it (*z*–*x*) and (**c**) in the transverse plane (*z*–*y*, at *x* = 0.5 *λ*_0_ = 0.4 μm behind the foil) at *t* = 7.6 *T*_0_ = 20.3 fs after the peak of the laser pulse reached the tip of the target. The electric field responsible for the TNSA mechanism is that generated by the electron sheath at the rear of the foil at *x* > 5.3 μm (**a**,**b**), whereas the field structure in front of the target generated by plasma currents is of minor importance for the proton acceleration. The one-dimensional graphs (green) illustrate the average of the electric field *E*_*x*_ in the range *x* ∈ [5.2, 10] μm, computed along the *y* or *z* direction (graphs at top) or along the *x* direction (graphs at the side), where the corresponding graphs in (**a**,**b**) have the same scales. The rings of the sideways pattern in (**c**) have a thickness of ≈*λ*_0_. The simulation parameters are the same like in Fig. 6(b).

First the void in the foil given by the implemented hemisphere alters the motion of the accelerated electrons at the rear side of the target. Due to the lack of positively charged ions in that gap the electrons are radially pulled in the outward direction and thereby dragged back by the ions of the surrounding flat foil. In this process they form arc-shaped structures in the electric field *E*_*x*_ normal to the target around the $z=0$ plane [Fig. 7(a)]. Note that the linear polarization of the pulse points in the *y* direction. These structures are superimposed to the classical TNSA sheath field, which is the only component present in the perpendicular plane [Fig. 7(b)] and is generated by the electron space charge. The electric field strength is more pronounced in the *y*–*x* plane at the edges [Fig. 7(a,c)]. The transverse cross section of the electric field at a distance of 0.5*λ*_0_ behind the foil [Fig. 7(c)] reveals the same pattern as the configuration of the trajectory origins in Fig. 6(b).

**Figure 8:**
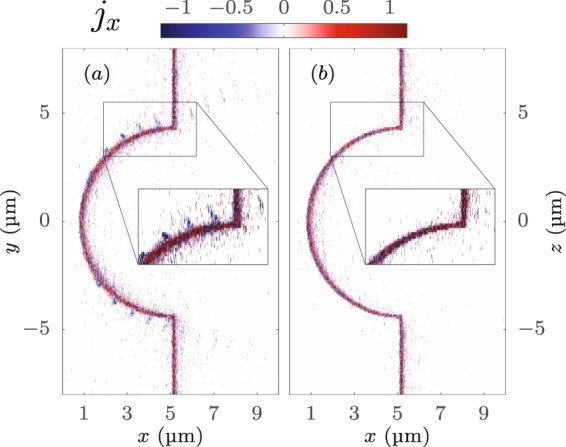
Lateral cross section of the current-density component *j*_*x*_ [MA/μm^2^] normal to the surface in (**a**) the plane of polarization (*y*–*x*) and (**b**) perpendicular to it (*z*–*x*) at *t* = 7.6 *T*_0_ = 20.3 fs after the peak of the laser pulse reached the tip of the target. The magnified inset (**a**) reveals electron bunches of distance ≈*λ*_0_ drawn out of the target, which do not exist in (**b**). The simulation parameters are the same like in Fig. 6(b).

Secondly, there are additional electrons directly extracted out of the hemisphere by the laser electric field. Once they move freely in vacuum, they are pushed forward through the foil by the $\overrightarrow{v}\times \overrightarrow{B}$ force, which becomes substantial for relativistic intensities, as their momenta scale like $\propto {a}_{0}^{2}$ compared to the transverse direction (∝*a*_0_). This effect of the so called direct laser light pressure acceleration (DLLPA) was observed by *Gaillard et al*. before^24^ for μm-thick targets, in the regime of classical TNSA. Depending on the surface conductivity, the incoming pulse will be reflected, resulting in a modulated electromagnetic field responsible for the forward motion of the electrons in the locally longitudinal electric field^32^. Taking a closer look into the extraction process of those electrons we see a strong dependence on the laser polarization direction. In the *y*-direction – which is the direction of the laser electric field – the electron current density component normal to the target *j*_*x*_ in the corresponding *y*–*x* plane is significant [Fig. 8(a)], while non-existent in the perpendicular *z*–*x* plane [Fig. 8(b)]. This can be understood envisioning the projections of the hemisphere. It is much easier to extract electrons in the direction normal to the ultra-thin target, which is the case along the *y* axis, compared to the parallel extraction which would be the case along the *z* axis. The blue bunches, separated by distances of ≈*λ*_0_, indicate forward moving electron packs outside of the target. These occur periodically at the edges between the shell and the surrounding foil and move through the target to the rear side where they contribute to a higher electron density. Consequently, the electric field *E*_*x*_ has its maximum at these specific regions and exhibits concentric rings of thickness ≈*λ*_0_ [Fig. 7(a,c)] due to the cyclical electron bunch generation and transportation by the laser pulse.

**Figure 9:**
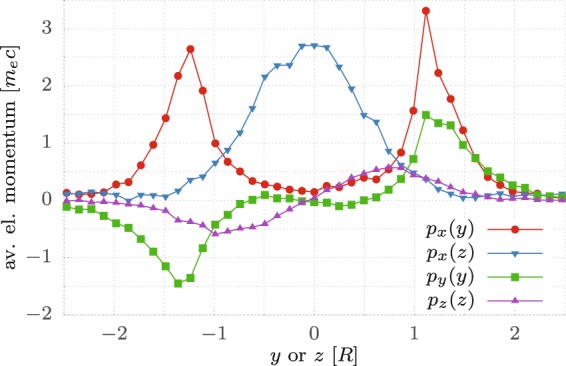
Averaged electron momenta over the transverse direction *y* or *z* in units of the hemisphere radius *R* = *D*/2 = 4.4 μm. The time is *t* = 6.6 *T*_0_ = 17.6 fs after the laser pulse impinges on the target. In the longitudinal direction (*x*), only electrons at least 0.5 *λ*_0_ = 0.4 μm behind the foil were taken into account to filter out the offset created by the electron sheath at the back of the foil [see Fig. 7(a,b)]. In the transverse direction (*y* or *z*) the momenta were averaged along the direction perpendicular to the regarded coordinate. The simulation parameters are the same like in Fig. 6(b).

A further proof of the above presented theory is the analysis of the phase space of the electrons from the hemispherical target (Fig. 9). The electrons situated behind the target solely due to heating would have a symmetric outward directed momentum $p(r)$ as described by the point-symmetric purple curve ${p}_{z}(z)$, giving evidence that electrons have zero transverse momentum in the centre and the strongest momentum at the edge ($r=R=D/2$, where *R* is the shell radius). The momentum in the polarization direction ${p}_{y}(y)$ (green curve) is point-symmetric as well, however it is essentially zero for $|y| < R$. It has higher maxima which lie slightly outside at ≈$1.25\,R$. This can be attributed to the above-mentioned effect, that electrons are radially pulled out of the hemispherical part of the target at which the electric field vector $\overrightarrow{E}$ of the pulse has a component normal to the surface. The forward momentum ${p}_{x}(y)$ has its peaks at the same positions ≈±$1.25\,R$ (red curve), whereas in the perpendicular view ${p}_{x}(z)$ the peak of same magnitude is at the centre $z=0$ (blue curve). This describes the currents that are strongest at the edges of the hemisphere in the *y*–*x* plane and become weaker for $|z| > 0$ (Fig. 8), as the component of $\overrightarrow{E}$ normal to the surface becomes smaller.

For the case of a flat target there is no additional gain in ${p}_{x}(y)$, since the electrons cannot be dragged out of the target by the laser electric-field and do not gain energy by the $\overrightarrow{v}\times \overrightarrow{B}$ force. In this case ${p}_{x}(y)$ and ${p}_{x}(z)$ are of similar magnitude and axisymmetric with respect to the centre, resulting in an almost uniform forward momentum of the electrons across the focal spot. This forms an even sheath-field on the target rear surface, which transfers onto the proton beam profile, giving a uniform acceleration as shown in Fig. 6(a).

In order to further study the intensity dependence over a wider range, additional simulations for irradiation intensities of 2 × 10^19^ W/cm^2^ (${a}_{0}=3.1$) and 1 × 10^21^ W/cm^2^ (${a}_{0}=21.6$), as shown in Fig. 5, were carried out. The obtained proton energies are 1 MeV (flat target) and 4 MeV (hemispherical target) in the low intensity case for a plane wave (see the method section) and 46 MeV (flat target) and 70 MeV (hemispherical target) in the high intensity case for a focussed laser beam 4.4 μm off-centre of the hemisphere. Both simulations imply a factor of 3.5 (low intensity) and 1.5 (high intensity) more energy for the case of the hemispherical target.

**Figure 10:**
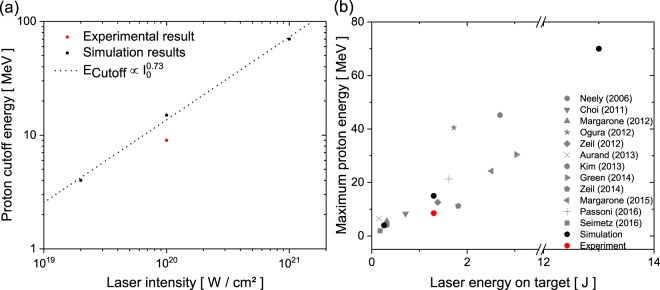
(**a**) Scaling of the maximum proton energy as a function of the laser intensity: Simulation results and a fit yielding a ${I}_{0}^{0.73}$ scaling. (**b**) Comparison of the achieved proton energy with respect to the laser energy on target with other experiments. (*Neely et al*.^44^; *Choi et al*.^45^; *Margarone et al*.^46^; *Ogura et al*.^47^; *Zeil et al*.^48^; *Aurand et al*.^21^; *Kim et al*.^49^; *Green et al*.^50^; *Zeil et al*.^51^; *Margarone et al*.^52^; *Passoni et al*.^53^ and *Seimetz et al*.^54^).

A closer look into the particle trajectories reveals that the DLLPA mechanism supports the acceleration process at both additionally studied intensities and can therefore be attributed as the driving mechanism over at least two orders of magnitude in intensity. The results for the three different intensities can be used for a rough intensity-scaling, as shown in Fig. 10(a). From the three data points it can be deduced that the proton cut-off energy scales with ${E}_{{\rm{cutoff}}}\propto {I}_{0}^{0.73}$. The red point marks the experimentally achieved energy.

Comparing our finding to the intensity scaling of TNSA with ${E}_{{\rm{cutoff}};{\rm{TNSA}}}\propto {I}_{0}^{0.5}$ and the scaling of RPA with ${E}_{{\rm{cutoff}};{\rm{RPA}}}\propto {I}_{0}$, we find ourselves in a hybrid regime, which was observed by other groups before, e.g. *Higginson et al*. holding the current energy record of about 94 MeV protons^23^. On the one hand TNSA is still the driving mechanism, boosted by the additional electrons of the DLPPA process, while an onset of a collective mechanism like RPA or its precursor, the hole-boring (HB) regime is observed. Since we use linear polarized light, causing a heating of the electrons, even for the highest intensities RPA does not become the dominant mechanism. Indeed, we observed in the simulation that for a thickness of 110 nm the target is exploded by the laser, which is likely due to the violent removal of the electrons by the linear polarized field. This limits on the one hand the collective acceleration effects and removes on the other hand the electrons necessary for building up the sheath field for TNSA. Redoing the simulations for thicker targets, reveals a suppression of a direct explosion but at the same time a decrease in the maximum proton energy since the target becomes too thick for the collective acceleration.

In Fig. 10(b) we compare our experimental- and simulation results to a number of studies which were done at different multi-Joule laser-systems. This reveals that our experimental results are in a good agreement and the plausibility of the simulated scaling. In a direct comparison to the previous DLPPA results obtained by *Gaillard et al*.^24^, they achieved a maximum proton energy per Joule of laser energy of 0.82 MeV/J, while we retrieved 6.5 MeV/J. Even taking into account that the overall ion acceleration for the high-energy lasers with tens of Joule of output energy and several 100s of fs pulse-duration is slightly less efficient in terms of MeV/J, compared to ion acceleration by multi-Joule systems at 10s of fs pulse-duration, as can be seen e.g. in the comparison by *Borghesi et al*.^33^, this is a great enhancement. For the simulations the comparable energy gain in our studies was 11.5 MeV/J for *a*_0_ = 3.1, 13.5 MeV/J for *a*_0_ = 6.2 and 5.4 MeV/J for *a*_0_ = 21.6. Summarised, this demonstrates that the DLLPA mechanism is very well applicable at a variety of multi-Joule laser-systems.

Another issue is the shape of the beam profile for the different targets. As shown in Fig. 3(a), the profile of the proton beam does not change using the hemispherical- and the flat target, while other groups report on changes in beam shape or divergence using tailored targets^34,35^. To our understanding, the difference is the geometrical scale and target thickness. While in the above-mentioned studies, the formation of the overall electron sheath field at the target rear side is manipulated, which imprints directly on the proton beam profile, this effect happens in our case on a much smaller scale. Looking at snapshots in the PIC simulation of the proton trajectories from the hemispherical part at time steps of a few hundreds of femtoseconds, some protons originating from the hemisphere radially move inwards the geometrical centre, driven by the local sheath field along the inner wall of the hemisphere. Due to their repulsive charge these particles cross each other and diverge again, with a large opening angle. Since these particles are only a small portion of all particles being accelerated, the effect is not dominant and washed out over the macroscopic distance up to the detector. The majority of protons is accelerated by the surrounding sheath field on the flat part of the target, which is, independently from being boosted by DLLPA, homogeneous and therefore not imprinting a different divergence on the protons. It remains the question of the optimal target geometry. The fundamental requirement for this process is that a part of the target structure needs to be parallel to the laser direction and perpendicular to the electric field vector, for example realizable by elongated structures like ellipsoids, cuboids or wires. At the same time the electric field on those structures should be sufficiently high to be able to extract and accelerate the additional electrons. Therefore, structures must be on the order of the laser focal diameter or smaller. The targets used in this study have a centre-to-centre distance so high that the electrons extracted from the shell-structures do not significantly interfere with the neighbouring hemispheres or prevent the laser pulse from propagating down to the bottom of the target. In the case of micro-^36^ and nanowires^37^ it was shown that for higher densities of the wires the surrounding gaps fill with plasma. Depending on the extent of the filling, this can either aid the absorption of laser light into hot electrons or leads to reflection of the incoming laser pulse if a critical density layer has been formed. It might therefore be beneficial for the TNSA part of acceleration to engineer the filling factor in a way to additionally profit from a filling within the vacancies of the target. While for the sake of studying the isolated effect of the tailored structure, a large separation in between the hemispheres was chosen, in further applications this distance can be minimized, allowing an easier experimental alignment of the target without the need of adjusting it onto a specific single structure.

## Conclusion

We demonstrated the benefit in energy gain by using ultra-thin hemispherical targets compared to flat targets. An energy increase by a factor of 1.8 was measured. By means of numerical simulations, this increase could be identified as protons originating from an area of about 5 μm from the flat part of the target around the hemisphere. It turns out that additional electrons extracted out of the hemisphere which are pushed through the target by the laser pulse enhance the accelerating sheath field for the protons from the rear side of the target. This leads to an energy increase of the protons. While before, this effect of direct laser light pressure acceleration (DLLPA) was only observed at high-energy lasers for μm-thick targets, we experimentally achieved an energy gain with only 1.3 J of laser energy which corresponds to a maximum proton energy to input laser energy ratio of 6.5 MeV/J. We proved in simulations that by a simple target geometry DLPPA can be scaled over a wide intensity range achievable at many of today’s already existing laser-systems. This wide range scalability of the process holds promise for many applications since it can be universally applied, making it a potent candidate to help reaching necessary particle energies in hadron therapy, as a driver for fast ignition in inertial confinement fusion or for probing magnetic and electric fields with high spatiotemporal resolution.

## Methods

### Target preparation

The targets were prepared as follows: Templates for the targets were defined in a 10 μm thick polystyrene layer (PS, 350 kg/mol, Sigma-Aldrich) on 1 mm thick glass. The PS-layer was structured by thermal nanoimprint^38,39^. A 2 × 2 cm^2^ negative stamp from OrmoStamp (micro resist technology GmbH, Germany) on 1 mm thick glass^40^, featuring hemispherical structures (diameter ≈8 μm, pitch ≈35 μm), was used. The surface of the OrmoStamp stamp was provided with an anti-sticking layer^41^. A pressure of 10 MPa was applied to transfer the stamp structures into the PS layer at 190 °C within an imprint time of 5 min. On these positive structures from PS a layer of parylene (${[{{\rm{C}}}_{8}{{\rm{H}}}_{8}]}_{n}$) was deposited via chemical vapor deposition (CVD). Similar to our previously used method^21,42^, we floated the parylene on our target mounts. In this case, we used a mixture of toluene and acetone (1:1), which dissolves the PS but not the parylene within 24 h.

### Diagnostics

Two different diagnostics were used, either a Thomson parabola spectrometer (TP) or a stack of radio-chromatic films (RCF)^43^. The TP was coupled to a micro-channel plate (MCP) detector, which was set up to detect protons and ions in the energy range between 0.3 and 30 MeV in the forward direction related to the laser axis. The RCF stack (Gafchromic™ HD-V2), wrapped in 14 μm aluminium foil, was placed 5 cm behind the target to reveal the proton beam profile (E_14μmAl_ > 1 MeV). The stack could be moved in order to record several shots in one run.

### Particle-in-cell simulations

The laser pulse of ${\lambda }_{0}=800\,{\rm{nm}}$ wavelength was modelled as a focussed pulse with a focal spot of 7 μm diameter (FWHM) for the normalized laser amplitudes of ${a}_{0}=6.8,21.6$ (corresponding to *I* = 1 × 10^20^ W/cm^2^, 1 × 10^21^ W/cm^2^) and as a plane wave for ${a}_{0}=3.1$ (corresponding to *I* = 2 × 10^19^ W/cm^2^). The temporal profile is a Gaussian function. For the size *x* × *y* × *z* of the simulation box the measures *x* = 12.1 μm and *y* = *z* = 24 μm (plane wave) or 27.2 μm (focussed pulse) and grid steps Δ*x* = 9.2 nm and Δ*y* = Δ*z* = 72 nm were used. In case of the simulations with a shifted laser pulse the lateral size *y* = *z* was increased to 36 μm to fit the pulse into the box. In the high intensity case *a*_0_ = 21.6 the gridsteps were Δ*x* = 7.4 nm, Δ*y* = Δ*z* = 79.6 nm and the longitudinal size *x* was increased to 20.1 μm to have enough space for acceleration. The simulation time was 150 laser cycles, corresponding to 400 fs, with a timestep of Δ*t* = 0.016 fs. The target with ion number density n_C_:n_H_ = 8:8, similar to the parylene target used in the experiment, was assumed to be not pre-ionized at a density of ${{\rm{n}}}_{{{\rm{C}}}_{8}{{\rm{H}}}_{8}}=6.4\times {10}^{21}\,{{\rm{cm}}}^{-3}$. For each of the hydrogen and carbon atom species we used two macro-particles per cell. A 110 nm thick layer, either a flat target or a hemisphere (*R* = 4.4 μm) surrounded by a flat area, was placed in the box and irradiated by the laser pulse at normal incidence from the convex side of the shell. To ensure that the cut-off energy retrieved from the low-populated high-energy tail of the spectra given in Figs. 4 and 9 are based on a representative particle statistic, we added an exponential fit over the full spectral range, which matched the cut-off energy observable from the graph with an error of less than 10% in all cases.

## Data Availability

The data that supports the findings of this study are available from the corresponding authors upon request.
